# Students of physical therapy. A comparative study of student profiles at a college and university in Israel

**DOI:** 10.1100/tsw.2006.104

**Published:** 2006-05-05

**Authors:** Nitza Davidovitch, Josepha Danziger

**Affiliations:** Department of Academic Development, Academic College of Judea and Samaria, Ariel, Israel

**Keywords:** physical therapy, medical education, human development, disability, student profile, regional college, Israel

## Abstract

This study focuses on the attributes of students of physical therapy in order to compare the profiles of students of physical therapy (PT) in two institutions of higher learning in Israel: Ben Gurion University (BGU) and the Academic College of Judea and Samaria (ACJS). This study focuses on a department where studies have an occupational/applicative/practical orientation and high status in the higher education system. Findings of this study indicate broad similarities in the profiles of students at both institutions in relation to their age, family status, country of origin, number of siblings, parental education, and financial status of student family of origin. On the other hand, students at both institutions differed in terms of gender composition, students' employment status, the source of payment for tuition, and in their academic attainments prior to admission. Specifically, students of physical therapy at ACJS had lower academic achievements prior to their admission and reported having been rejected by other physical therapy programs. Students at ACJS placed higher importance on factors relating to the quality of instruction including teacher involvement, competitiveness, organization, control, and orientation to the study material. Students at BGU attributed greater importance to teachers' support. Findings support a thesis of a converging system of higher education in Israel, traditionally dominated by national universities and regional colleges, a relatively recent phenomenon.

## INTRODUCTION

Physical therapy aims to facilitate the rehabilitation of patients with an acute problem, the disabled and aged individuals in order to restore their maximum physical functioning, and prevent further disability with intervention as early as possible. This profession has transformed in recent decades from a purely therapeutic occupation to a scientific-research-based discipline that develops original bodies of knowledge, drawing from sources such as natural sciences, social sciences, and medicine. This profession focuses on physical diagnoses, electrical and mechanical treatments, as well as treatments based on radiation, water, or heat based on an understanding and knowledge of the human body in health and illness, taking into consideration mental and social factors that affect the lives of individuals. Physical therapists must have the ability to implement basic knowledge in the natural sciences, behavioral sciences, and medicine, and the ability to plan and execute physical therapy procedures in the diagnosis, treatment, and evaluation of patients. Physical therapists are also required to have understanding and skills in the art of interpersonal communications, an ability to address the ethical standards of the profession, and an ability to work in a multidisciplinary team of professionals. The relatively recent academic approach of the profession has promoted research in the field and supported the development of a comprehensive and in-depth foundation for multidisciplinary training, setting a high academic standard for students and professionals.

## Physical Therapy Programs in Israel

In Israel, physical therapy is of unique importance in view of the large numbers of casualties of war and terrorist attacks, on one hand, and of a high rate of road accidents, on the other. These two factors have created a large population of relatively young individuals and children who require extended holistic treatment and care.

In Israel, the discipline of physiotherapy was developed in the 1950s and, as a response to the polio epidemic in 1953, the School of Physical Therapy affiliated with Assaf Harofeh Hospital was established. The Wingate Institute School of Physical Therapy was established in 1963 in response to a lack of certified physical therapists needed for rehabilitation and physical therapy treatments of bed-ridden patients in their homes or in hospitals. The Sheba Hospital School of Physical Therapy was established in 1976 by the Ministry of Health to train physical therapists for work in a new area: community-based identification of needs and service provision. The aim was to provide services to all age groups, in all states of health, ranging from full health to chronic illnesses requiring confinement. In the 1980s, physical therapy transformed from an occupation to a research discipline, BPT (Bachelor of Physiotherapy). In 1982, the Department of Physical Therapy was established at the Sackler School of Medicine of Tel Aviv University, while those at the Ben Gurion University of the Negev, Haifa University, and the Academic College of Judea and Samaria followed in 1983, 1984, and 2001, respectively. A National Registry of physical therapists was established by law and this certification offers graduates the opportunity to work in a broad range of institutions, including general hospitals, outpatient clinics, neurological rehabilitation, geriatric rehabilitation, care for children with developmental disability, and special education schools. Despite the fact that all academic programs are authorized and standardized by the Higher Education Commission, there are some differences in the attitudes and focus of each program in the area of field-work relative to academic studies.

Departments of Physical Therapy at institutions of higher learning in Israel are affected by global changes introduced by the Israeli system of higher education in response to challenges of autonomy, individualization, and democracy, enhanced by a growing immigration population and the growing specialization of the labor market. Today, society expects academic institutions to be extensively involved in social problems with a focus on academic teaching and research programs. Society expects a system that meets the national needs of professional training to support national progress and provide the professional, technological, and intellectual infrastructure for improving efficiency, effecting savings, and modernizing the economy.

In response to the changing demands, the goals of higher education in Israel have undergone revolutionary changes on many levels[1], from the impartation of higher education for its own sake to a vocation orientation designed to provide practical and professional training; from the view of education as a goal to the view of education as a means; from a view of the goal of education as expanding knowledge and supporting discovery and exploration to a view of higher education in service of society, underlined by attempts to politicize the higher education system; from a trend of increased focus and specialization to a multidisciplinary focus; and finally, from an emphasis on excellence in studies to an emphasis on the value of equality for all[1]. These trends have driven the diversification of institutions and growth in the number of students in postsecondary education and, more importantly, created a structural transformation of the higher education system in Israel.

One significant structural change has been the emergence of regional colleges, which developed in a short period from independently operating, small-scale colleges that offer local study programs into full-scale academic colleges with all the administrative changes implied in such a shift. The amendment to the Higher Education Law[2] regulated the status of colleges as academic institutions and formally equalized the status between degrees awarded by the colleges and the universities. On one hand, regulations[3] require colleges to adopt university-like priorities, but on the other hand, concerns are to preserve the currently existing university format, which may create dialectic between the needs of society and the needs of students. This moral collision dictates that regional colleges adopt academic and organizational patterns of operation to reflect a stance of social responsiveness. Gradually, as the stigma of colleges as second-class universities has declined, the status of degrees awarded by regional colleges is perceived as legitimate and worthy.

The ACJS (Academic College of Judea and Samaria) was founded in the late 1980s as a local college by serving as an extension of Bar Ilan University. In the second half of the 1990s, the college became the largest public academic college in Israel and achieved independent status in 2002–2003, accredited by the Commission for Higher Education to award academic degrees. From its establishment, the college's academic activities were assumed to be of a "university-orientation" nature. The college combined both facets of higher education: on one hand, it invested intensive efforts to create a national demand for the college and its degrees by combining research and teaching, and on the other hand, it maintained and developed its local functions as a community service. Although milestones in the course of the college's development corresponded to those of other academic institutions in Israel, the ACJS was the only one of the five local colleges sponsored by Bar Ilan University to attain independent academic status. Since its establishment, the college has continuously promoted university-oriented activities and the fruits of these efforts have gradually matured over the years in research, in the diversification of its faculties and departments, and in the increasing number and standards of its academic faculty. The ACJS is also similar to universities in its administrative-organizational structure, and in its procedures for recruiting senior faculty members.

The Ben Gurion University of the Negev (BGU), established in the 1970s, is a fine example of a university institution that developed in response to local needs and its activities had both local and national repercussions. In many ways, the development of this university parallels the growth of local colleges. From its inception, BGU was not merely "one more university" detached from its social environment secluded in an ivory tower of research, but a sense of involvement in social and community issues and their relevance to study and research programs has accompanied the university since its foundation[4].

## METHODS

In the present study, we compared students of physical therapy at schools representing two different types of academic institutions. We examined the links between study variables (students' personal and family attributes, their academic background, motives for enrollment, self-efficacy, and preferred aspects of academic-social climate) at ACJS and BGU. We projected that a comparative analysis of the profiles of physical therapy students at two different institutions of higher learning in Israel, and the exploration of inter-relations between profile indices (personal and family data, academic background, motives for enrollment, self-efficacy, and academic-social climate), would provide an important key to understanding key development scenarios of the physical therapy departments at different types of academic institutions in Israel[5]. One question was the extent to which physical therapy studies were consistent with a binary academic system, characterized by a clear division of labor between various types of academic institutions, each targeting different population groups, or whether physical therapy programs in different academic institutions target similar student needs.

## Study Population

The study population comprised a total of 115 students from the departments of physical therapy at ACJS (82) and BGU (33) who responded to the survey. The students completed a questionnaire containing items on their personal, academic, and family background, as well as their motives for enrollment in the program. They also completed a self-efficacy survey and a questionnaire on their perceptions of the academic-social climate at their respective institution. Students were coded by institution and by year in the program. We included questionnaires of all participants, although several students failed to complete all questionnaire items.

## Tools

To obtain information on students and their perceptions, we developed a questionnaire comprised of the following five sections: personal and family details, academic background, motives for enrollment[6], self-efficacy[7], and academic-social climate preferences[8]. Our aim was to identify and compare the perceptions and preferences of students of physical therapy attending the two institutions under investigation, as well as their background details.

## Research Questions

1. Do physiotherapy (PT) students from the two institutions differ in their personal and family attributes?
2. Do PT students differ in their academic background?
3. Do PT students differ in their motives for enrollment in the respective programs?
4. Do PT students hold different preferences of aspects of their academic-social climate?
5. Do PT students differ in their self-efficacy levels?
6. Do PT students differ in their personal and family attributes, their motives for enrollment, academic-social climate preferences, and their perceptions of self-efficacy, by year in the program?
7. Do PT students differ in their personal and family attributes, their motives for enrollment, their academic-social climate preferences, and their perceptions of self-efficacy, by gender?
8. To what extent did these variables jointly explain students' enrollment? Are there any other factors that potentially effect enrollment in either institution?

## Research Instruments

We used the following four questionnaires in this study:

1. Personal, family, and academic background information[6] — This section of our study[5] included 22 items relating to students' background (country of origin, gender, family status, economic status of family, sources for tuition) and academic background (psychometric exam score and matriculation average).
2. Enrollment motives[6] — The questionnaire[5] was comprised of 15 items relating to several groups of factors influencing the decision to apply to the PT program:

a. Personal-financial factors: flexibility of schedule, convenient options to cover tuition fees, family direction, selection made by employer, proximity to place of residence, available dormitories
   b. Admission: acceptance to the current program despite having had applied to several institutions, more lenient admission criteria
   c. Institution: less-demanding program, more pleasant social atmosphere, higher standard of program, impressive academic facilities (libraries, laboratories), prior positive experience with the institution
3. Academic-social climate question — This questionnaire was developed by Moos[8] and adapted and translated into Hebrew by Levinson[9]. The questionnaire was originally designed for elementary and high school students and was, therefore, adapted to academic institutions[5]. Items appropriate for students in higher education institutions in Israel were selected. The questionnaire contains 90 Yes/No items reflecting 8 categories of factors: involvement, connections, teachers' support, target-oriented, competition, organization, teachers' control, and diversity. Range of internal consistency for the original questionnaire was $\alpha = 0.45–0.78$.
4. Self-efficacy questionnaire — This questionnaire is based on a questionnaire developed by Gibson and Dembo[10] and translated into Hebrew by Rich[11]. The original questionnaire, which related to teachers' self-efficacy, was adapted to the present study to relate to students' self-efficacy. Self-efficacy items were classified into four categories by content[5]: ability to develop independent study methods, ability to exert individual effort, family's contribution to academic abilities, and ability to establish contact with other students. A principal components' factor analysis with vari-max rotation was performed. Four factors explained 51.6% of the variance in the present study.

Based on the factor analysis, we calculated average scores for each participant, ranging from 1–6, with higher averages reflecting a higher level of self-efficacy. Score reliability ranged between 0.55–0.65.

## Statistic Analysis

A self-efficacy score was constructed on the basis of the 14 questionnaire items. To examine differences in self-efficacy perceptions by institution and gender, we performed a two-tailed t-test on independent samples.

We constructed several indices based on the academic-social climate questionnaire: academic-social involvement, academic-social connections, teachers' support, competitiveness, organization, teachers' control, clarity of rules and regulations, control over rules, innovativeness, orientation to study material

We performed two-tailed t-tests on independent samples to examine the differences between the various score of academic-social climate. We performed bi-directional analyses of variance to examine differences in scores by year in program.

To examine the differences between institutions, we performed $\chi^2$ and bi-directional variance analyses. We calculated Pearson coefficients for each participant and each institution to examine the connections between the study variables. We also performed Fisher's z analyses to examine the difference between correlations in each institution. To explain the extent to which the variables jointly explain the variance in both institutions, and whether the same variables affect students' variables in both institutions, we performed hierarchical regression analyses separately for each institution. Significant results attained significance at a $p < 0.05$ level.

## RESULTS

- **Students' personal/family attributes** — We found a correlation between gender and institution ($X^2(1) = 6.54$, $p < 0.05$). At BGU, only 9.4% of the PT students were male, compared to 32.9% of the PT students at ACJS. We also found a correlation between students' employment status and institution ($X^2(2) = 7.45$, $p < 0.05$). Of all students responding to this item, all PT students at BGU are employed, while 14.6% of all responding PT students at ACJS are unemployed.

- **Family status** — Most students (80%) in both institutions pay their tuition fees with the help of their parents or other relatives, while merely 21.7% of all students worked to cover their tuition fees. However, almost one-third of all PT students at BGU received scholarships, compared to a mere 7.3% of PT students studying at ACJS.
- **Academic background** — Our second research question concerned the differences in the academic background of PT students at ACJS and BGU. The following differences between these two groups of students were found in psychometric scores and matriculation averages. Significant differences in psychometric scores on admission were found by institution ($t(98) = 8.85$, $p < 0.001$). Average psychometric score of BGU students on admission were higher than the scores of their counterparts at ACJS (M = 676.03 and M = 612.21, respectively). Significant differences in average matriculation grades on admission were found by institution ($t(92) = 4.22$, $p < 0.001$). Average matriculation grades of BGU students were higher than those of ACJS students (M = 100.78 and M = 95.72, respectively).
- **Motives for enrollment** — Our third research question concerned differences in motives for enrollment in the respective PT departments. We found differences in students' motives for enrollment by institution. Major motives for students in the BGU PT therapy included a pleasant social atmosphere (93.9%) and high academic standards (84.8%). In contrast, the major motives of students at the PT program at ACJS included acceptance (73.2%), admission requirements (56.1%), and rejection by other institutions (51.2%). In summary, prior to their acceptance, BGU students placed a greater weight on a pleasant social atmosphere and high academic standard, while ACJS students were concerned with admission requirements, rejection by other institutions, and acceptance to ACJS.
- **Perceived academic-social climate** — Our fourth research question concerned differences in perceptions of PT students regarding the academic-social climate of their institution. We constructed nine indices on the basis of the academic-social climate questionnaire. Index scores ranged from 1 to 5, with 1 representing a less preferred aspect of the academic-social climate, and 5 representing a more preferred aspect of the academic-social climate. We found significant differences between the various indices of academic-social climate, by institution. The following indices received higher scores by ACJS students: involvement, competitiveness, organization, teachers' control, control over rules, and orientation to study material. Teachers' support was the index that was rated higher by BGU students. In contrast, college students attributed more weight to professors' involvement, competitiveness, organization, teachers' control, control over rules, and teachers' orientation to the study material. In summary, ACJS students attributed greater weight to factors relating to the practice of teaching and its complex facets.
- **Perceived self-efficacy** — The fifth research question concerns the differences in perceived self-efficacy of PT students at both institutions. We found significant differences in self-efficacy levels of students at both institutions ($t(110) = 2.49$, $p < 0.05$). Self-efficacy levels of PT students at the ACJS were higher than self-efficacy levels of their counterparts at BGU.
- **Examination of differences by year in program** — Our sixth research question concerned the differences in personal and family attributes, motives for enrollment, perceptions of the academic-social climate, and self-efficacy for PT students at ACJS and BGU, by students' year in the PT program. Most students paid their tuition fees with the help of their parents or relatives (80.0%), however, the longer the students were in the program, the greater the percentage of students who paid for their tuition using their own salary. We found the following differences in motives for enrollment, by year in program: first-year students placed greater emphasis on teachers' involvement, organization, teachers' control, control over rules, and teachers' orientation to the study material, compared to students at a more advanced stage in the program. First- and second-year students placed greater emphasis on a competitive atmosphere, compared to third- and fourth-year students. It was interesting to note that third-year students attributed greater importance to teachers' support, than second- and first-year students.

- **Gender-based differences** — Our seventh research question concerned differences in personal and family background variables, academic background, motives for enrollment, perceptions of academic-social climate, and self-efficacy of students at ACJS and BGU, by gender. Gender-based differences were found only in students' sources for tuition payment. More male than female students pay for their tuition using their own salary (M = 42.3 and M = 11.3, respectively) or external scholarships (M = 23.1 and M = 3.8, respectively).
- **Factors effecting enrollment** — Our last research question examined the extent to which all indices jointly explain students' enrollment in the ACJS and BGU, and whether there were factors that affected enrollment in either of these institutions. To examine the correlations between these indices and the background questionnaire, we calculated two-sided Pearson correlations. We found correlations between the following: number of siblings and competitiveness and control over rules (the greater number of siblings a student had, the greater importance they attributed to competitiveness and control over rules); the earlier students immigrated to Israel, the more they valued teacher control; the later students immigrated to Israel, the more they valued clarity of rules. To examine the variables discriminating between both institutions (the ACJS and BGU), we performed stage-wise discriminant analysis. The variables entered into the analysis were self-efficacy, general academic-social climate index, personal/family attributes (gender, number of siblings, country of origin, father's education, mother's education, marital status), and academic background (psychometric score and matriculation grade average). We found that two variables discriminate between 88.9% of the students at these institutions (Wilk's Lambda = 0.35, X2(4) = 76.07, $p < 0.001$): psychometric score, matriculation average, self-efficacy, and father's education. The remaining variables did not contribute to the discrimination between institutions.

## Summary of Results

- **Personal and family attributes** — Students at both institutions were similar in the distribution of their age, family status, country of origin, number of siblings, parental education, and financial status of student family of origin. On the other hand, students at both institutions differed in terms of gender composition, students' employment status, and the source of payment for tuition.
- **Academic background** — At these two institutions, students' psychometric scores and matriculation grades on admission differed significantly. ACJS students had significantly lower scores on admission.
- **Motives for enrollment** — Students at the institutions under investigation differed in their motives for enrollment. For BGU students, key motives included a pleasant social atmosphere (93.9%) and high academic standards (84.8%). In contrast, for ACJS students, key motives included admission (73.2%), lenient admission requirements (56.1%), and rejection by other institutions (51.2%).
- **Academic-social climate preferences** — Students at the institutions under investigation differed significantly in their preferences for elements of an academic institution's academic-social climate. ACJS placed importance on factors including involvement, competitiveness, organization, teachers' control, teachers' control over rules, and teachers' orientation to the study material, while BGU students attributed greater importance to teachers' support. In general, ACJS students placed greater emphasis in their preferences on the practice of teaching.
- **Self-efficacy perceptions** — Students at ACJS obtained significantly higher self-efficacy scores.

We found that PT students in both institutions shared the following attributes: findings indicated that the longer the students attended the program, the greater the numbers of students used their own salary to finance their studies. We also found differences in motives for enrollment and differences in academic-social climate preferences, by year in program. First-year students appreciate teacher involvement, organization, control, and orientation to the study material. First- and second-year students appreciate a competitive atmosphere more than third- or fourth-year students. Interestingly, third-year students attributed greater importance to teacher support than either first- or second-year students.

We found gender-based differences in the sources for tuition payments. More male students than female students finance their tuition by working and more male than female students finance their tuition through external scholarships.

In summary, of all the factors we examined, we found that the factors discriminating between students at these two institutions were psychometric scores, matriculation grades, self-efficacy, and father's education. The remaining variables did not contribute to any discrimination between these two institutions.

## DISCUSSION

In this article, we examined the development trends of universities and regional colleges in Israel, comparing student profiles of physiotherapy programs in very different institutions: the first, a university program established over 2 decades ago, founded specifically to fulfill the needs of the Negev region and the other, a relatively new institution, similarly established to cater to the needs of a specific regional population. The present study was conducted in the context of a dynamic national system of higher education in Israel. One of the most profound developments in this system in the past decade has been the establishment and increasing enrollment in regional colleges. In the past, a distinction was made between the missions, roles, and potential offerings to students in colleges and universities. These are well documented and defined.

The developmental trends of Israeli universities and colleges point to the growing similarity of these two types of institutions. The present study examined the connections between student profiles and their sense of self-efficacy in their studies and the nature and academic-social climate of the academic institution they attended. We also examined the connection between these factors and perceived ability to succeed in their studies. The present study may be considered a preliminary test case for an examination on broader sections of student populations. In contrast to arguments that claim that the two types of higher educational institutions in Israel cater to two distinct student populations, the findings of this study reinforce our assessment of the higher education system in Israel as a monistic system, indicating the unification of the institutions in the system without eliminating the unique nature of each institution. The educational system in Israel, including the system of higher education, constitutes a tool of social equality in the hands of policymakers, offering additional support of the unification trend emerging between institutions that award undergraduate degrees. However, unification does not imply the obliteration of the unique characteristics of the population of each institution, but rather a unique perspective on these factors and their establishment as a significant means of social response.

The pattern of internal connections of student variables is similar and, in some cases, identical in both institution types. Although a specific coefficient or average may be higher in one institution, the general structure is reflected in both. In other words, despite the slight differences in specific variables, the present study confirmed an overall similarity between these two institutions – a university and a college. Thus, these findings are a preliminary indication that universities and colleges in Israel are not separate species, but rather distinct expression of a structure of connections that is typical of the structure of academic institutions in general, and the differences in specific indices reflect the known diversity in the system under investigation. From this vantage point, it appears justified to expect a monistic system, notwithstanding differences between individual institutions.

The existence of a monistic system is conditional on the sustainability of its parts. If certain parts consistently under-perform compared to other parts, the system cannot survive unless it transforms into a binary system. Therefore, universities may have organizational advantages over colleges, but colleges also may offer their own advantages in the form of a more supportive practice of instruction. The case of the Physical Therapy Department at ACJS reinforces the monistic scenario. In our opinion, this scenario will also apply to other colleges. We believe that this scenario will emerge in the absence of any central intervention by national authorities, especially through budgetary means. A change in the trends and a shift to a system reflecting binary characteristics will occur only if the budgetary factors change the methods used to budgetary universities and academic colleges.

The findings of this study should lead to comparative studies between types of academic institutions and fields of study: comparing of the nature of study programs at universities and colleges, studies on university and college graduates, comparisons of the achievements of students in universities and colleges enrolled in parallel programs and courses. These differences are important if we are to understand the role of the different types of institutions in the higher education “market”, the competition (either actual or potential) between these institutions, and the weaknesses and the strengths of each type of institution.

The findings of the present study are a preliminary indication of a trend toward change, and towards the obliteration of the dual structure of universities and colleges. These changes have also undermined universities’ exclusivity in certain areas noted in the past. We believe that there will be differences in quality between all academic institutions, universities and colleges, and colleges should be granted the appropriate conditions for competition.

## ACKNOWLEDGMENT

We are grateful for the collaboration with the Department of Physical Therapy, Recanati School of Allied Professions in the Community, Faculty of Health Sciences, Ben Gurion University of the Negev, Beer-Sheva, Israel on the data collection.
